# Quality-of-Life Impairment among Patients with Hidradenitis Suppurativa: A Cross-Sectional Study of 1795 Patients

**DOI:** 10.3390/life11010034

**Published:** 2021-01-08

**Authors:** Piotr K. Krajewski, Łukasz Matusiak, Esther von Stebut, Michael Schultheis, Uwe Kirschner, Georgios Nikolakis, Jacek C. Szepietowski

**Affiliations:** 1 Department of Dermatology, Venereology and Allergology, Wroclaw Medical University, 50-368 Wroclaw, Poland; piotr.krajewski@student.umed.wroc.pl (P.K.K.); luke71@interia.pl (Ł.M.); 2 Department of Dermatology, Faculty of Medicine, University of Cologne, 50923 Cologne, Germany; esther.von-stebut@uk-koeln.de; 3 Department of Dermatology, University Medical Center, Johannes Gutenberg University, 55122 Mainz, Germany; Michael.schultheis@unimedizin-mainz.de; 4 Dermatology Outpatient Office Dr. Uwe Kirschner, 55116 Mainz, Germany; info@hautarztpraxis-mainz.de; 5 Departments of Dermatology, Venereology, Allergology and Immunology, Dessau Medical Center, Brandenburg Medical School Theodor Fontane, 06847 Dessau, Germany; nikolakisgeorgios@gmail.com

**Keywords:** hidradenitis suppurativa, DLQI, quality of life

## Abstract

The chronic, inflammatory skin disorder hidradenitis suppurativa (HS) is associated well documented negative influences on patients’ quality of life (QoL). The aim of this study was to present more robust data on patients’ QoL impairment by demographic data and its correlation with well-known HS risk factors on a cohort of 1795 German patients. The instrument used for measuring QoL in this study was the Dermatology Life Quality Index (DLQI). Overall, patients reported a very large effect of HS on their QoL (mean DLQI: 13.2 ± 8.1 points), and 22% of the analyzed population even reported to consider the effect as extremely large. Women tended to experience significantly higher impairment than men (*p* < 0.001). QoL impairment correlated positively with pain (r = 0.581, *p* < 0.001), HS severity (measured by the International Hidradenitis Suppurativa Severity Score System (IHS4)) as well as Hurley. Neck involvement tended to decrease QoL significantly more than any other location (14.7 ± 8.3 points). This study confirms the enormous influence of HS on patients’ QoL in a large cohort. Knowledge of QoL impairment in such patients is crucial for proper understanding and holistic management of this disease.

## 1. Introduction

Healthy skin, due to its visibility, is an important organ for proper psychological functioning. Chronic disorders of the skin can have a devastating effect on the private and professional lives of those affected. Among the psychological problems resulting from chronic skin disorders, stigmatization, feelings of embarrassment, low self-esteem, stress, anger, shame and depression are reported most frequently [1]. Many studies confirm that visible skin disease may lead to suffering from bullying, sleep impairment and a decline in academic or work performance [2,3]. Chronic skin disorders, which are believed to cause a significant decrease in quality of life, include, among others, atopic dermatitis, psoriasis and hidradenitis suppurativa. Due to the affectation of multiple body areas, foul smell, purulent discharge and associated pain and/or pruritus, hidradenitis suppurativa (HS) has been documented to have a negative influence on patients’ quality of life (QoL) [4,5]. HS is also associated with a higher incidence of depression, insomnia or decreased sleep quality, fear of stigmatization and workplace challenges [6,7,8]. Moreover, HS lesions often affect anogenital areas and impair sexual activity and satisfaction [4,5]. To date, several papers regarding the influence of HS on QoL have been published. However, to the best of our knowledge, all this work was based on a limited number of patients. The aim of this study was to present more robust data on patients’ QoL impairment through demographic data and its correlation with well-known HS risk factors.

## 2. Materials and Methods

### 2.1. Study Group

This study included HS patients, who were selected for LAight^®^ therapy (a combination of intense pulsed light with radiofrequency) [9,10] in multiple dermatology outpatients’ centers in Germany between April 2017 and February 2020. Patients were only included if they gave informed written consent to the collection, storage and scientific evaluation of their routine data during LAight^®^ therapy in an electronic database hosted by LENICURA (the manufacturer of the medical device). All documented patients were examined by a doctor specifically trained on HS. The overall contraindications for LAight^®^ therapy included pregnancy, epilepsy and an implanted electrical device. Moreover, the treatment was not initiated in patients with extreme photosensitivity, implants within 10 cm of the lesioned skin, tattoos, piercings, skin neoplasms or previous treatment with fillers in the treated HS area. Routine demographic baseline data included gender, age, body mass index (BMI) and smoking habits. In line with guidelines for human studies and the World Medical Association Declaration of Helsinki, the anonymized data were then transferred by LENICURA to the consortium of experts of this paper for scientific evaluation.

The studied group consisted of 1795 patients (1152 females and 643 males). The patients were 40.0 ± 11.8 years old. The majority of subjects were reported to be smokers (55.6%), and 10.5% of the population smoked more than 25 cigarettes a day. The mean BMI of participants was 28.1 ± 6.2 kg/m^2^, qualifying the population as, on average, overweight. All patients had their HS severity assessed with the use of the Hurley staging system and affected body areas, and 634 subjects were additionally assessed according to the International Hidradenitis Suppurativa Severity Score System (IHS4) [10,11,12]. The worst pain in the previous 24 h was evaluated with the use of the Numeral Rating Scale (WP-NRS). In this 11-point scale, 0 implies no pain, and 10 implies the worst imaginable pain [13].

### 2.2. Quality of Life

In order to evaluate the actual influence of HS on the patients’ QoL, all subjects were asked to complete the Dermatology Life Quality Index (DLQI) questionnaire [14]. The DLQI is a simple, dermatology-specific, quality-of-life questionnaire. Since its development, it has been widely used in a variety of dermatoses, including psoriasis, atopic dermatitis and prurigo nodularis [15,16,17]. It is also a routinely used instrument in assessing improvements in QoL after novel treatments with biologics [18,19]. Until 2019, before the introduction of the Hidradenitis Suppurativa Quality of Life (HiSQOL) questionnaire [20], there was no HS-specific questionnaire; therefore, the DLQI was a crucial instrument used in the evaluation of HS patients’ QoL. This 10-item questionnaire was developed in 1994 by Finlay and Khan [21], and each item can receive an impairment degree on a 4-point-scale (0—not at all, 3—very much). Moreover, the questionnaire is divided into six subscales that assess the affectation of one of the QoL domains: symptoms and feeling, daily activities, leisure, work/school, personal relationships and treatment [21]. The maximum number of points achievable is 30 points, and the higher the score, the greater the impairment of QoL. The DLQI is to be interpreted with the following cut-off values for the effects on patients QoL: 0–1 = no effect at all; 2–5 = small effect; 6–10 = moderate effect; 11–20 = very large effect; 21–30 = extremely large effect [21].

### 2.3. Statistical Analysis

Statistical analysis of the obtained results was performed with the use of the IBM SPSS Statistics v. 26 (SPSS INC., Chicago, IL, USA) software. All data were assessed for parametric or nonparametric distribution. The minimum, maximum, mean and standard deviation were calculated. Quantitative variables were evaluated using the Mann–Whitney U test and Spearman’s and Pearson’s correlations. For qualitative data, the chi-squared test was used. Differences in total DLQI between patients with different HS severities according to Hurley stages were assessed with the Kruskal–Wallis one-way analysis of variance on ranks. All analyses were performed as two-sided tests with a significance level of 5%.

## 3. Results

**Figure 1:**
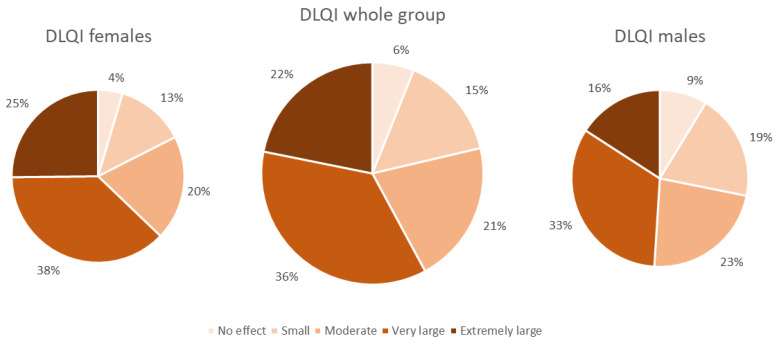
HS effect on patients’ QoL. The diagram is based on the DLQI cut-offs. HS—Hidradenitis suppurativa; QoL—Quality of life; DLQI—Dermatology Life Quality Index.

**Figure 2:**
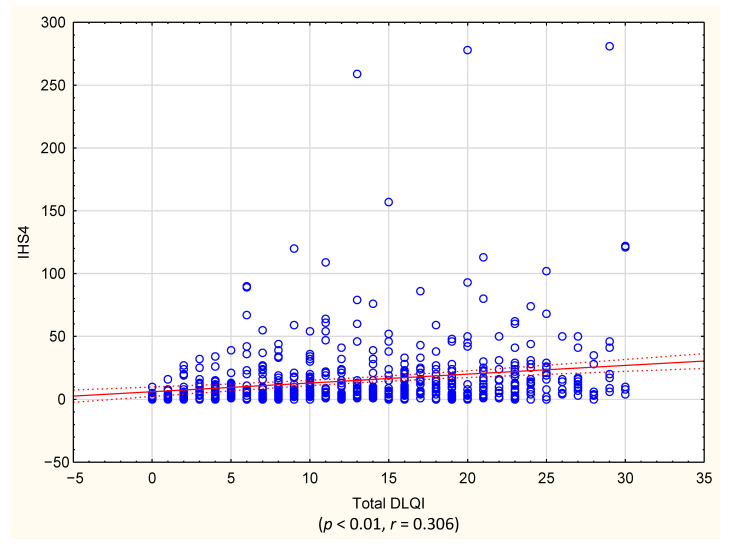
Correlation between disease severity (IHS 4) and quality-of-life impairment (DLQI). IHS4—International Hidradenitis Suppurativa Severity Score; DLQI—Dermatology Life Quality Index.

**Figure 3:**
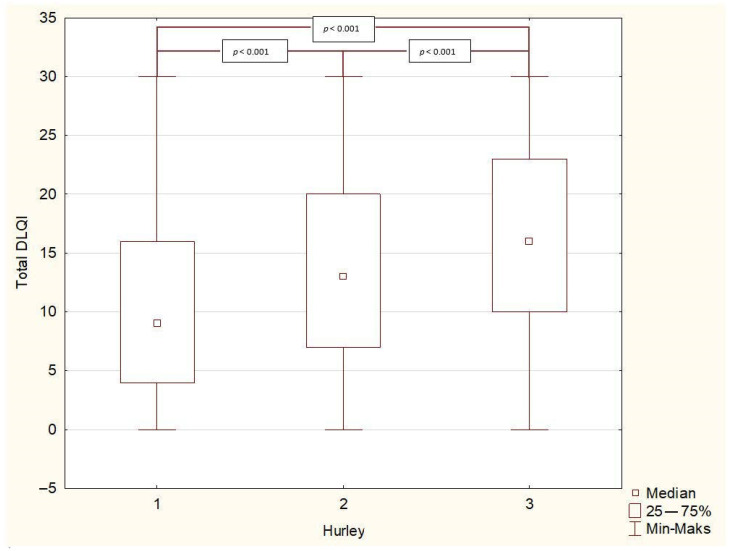
Total DLQI distribution among disease severity assessed with the Hurley severity score. DLQI—Dermatology Life Quality Index.

The perceived influence of HS on QoL in the studied group was found to be very large (mean DLQI: 13.2 ± 8.1 points). Female patients reported significantly higher impairment in QoL than males (14.2 ± 8.0 vs. 11.5 ± 8.0 points, *p* < 0.001). When categorized according to the DLQI cut-off values, a very large effect on patients’ QoL was observed most frequently (36% of patients), followed by extremely large (22%), moderate (21%) and small (15%). Only 6% of the patients reported that their HS had no effect on their QoL. The gender difference also manifested in different cut-off areas of the DLQI since the percentage of female subjects experiencing an extremely large effect on QoL was significantly higher than that of male patients (25% vs. 16%, *p* < 0.001) (Figure 1). This finding was also confirmed for the single items of the DLQI score as well as in the DLQI domains, in which, apart from Items 6 and 7 and the domains work and school, impairment was significantly higher for females than males (*p* < 0.01) (Table 1). HS severity had a significant influence on patients’ QoL, with a moderate correlation found between DLQI and IHS4 (r = 0.306, *p* < 0.001) (Figure 2). Moreover, a higher Hurley stage was associated with a greater negative impact on QoL in all DLQI-Items questions and domains (Figure 3). Interestingly, the number of affected areas seems to not decisively contribute to QoL impairment. There was a significant, but only weak, correlation (r = 0.1, *p* < 0.001) between affected areas and the DLQI and no significant difference in DLQI between single-area-affected patients and patients with multiple areas affected (*p* = 0.794). Regarding the affected areas, among subjects with spread disease, the highest life impairment was reported by those with neck involvement (14.7 ± 8.3 points, *p* < 0.001), and the lower extremities’ involvement was found to cause the lowest reduction in QoL (11.9 ± 7.9 points, *p* < 0.001). For the single-area affectation, there was no difference in the decrease in QoL between affected areas (Table 2). With respect to risk factors, QoL impairment showed a significant positive correlation with age and BMI; however, in both cases, the degree of correlation was weak (r = 0.1, *p* < 0.001). There was a significant difference (*p* < 0.001) in QoL impairment comparing smokers with nonsmokers (13.9 ± 8.1 and 12.4 ± 8 points, respectively); however, we did not find any correlation with the number of smoked cigarettes per day (detailed data not shown). Finally, concerning other patients’ reported outcomes, a strong positive correlation was found between mean DLQI and pain assessed by NRS (r = 0.581, *p* < 0.001).

## 4. Discussion

Hidradenitis suppurativa (HS) is a painful, chronic, multifactorial and progressive inflammatory skin disease of the pilosebaceous unit. It predominantly affects intertriginous zones of the body, including axillae, groins, buttocks or the submammary region in young adults, and leads to the development of inflammatory nodules, abscesses, sinuses and scarring [22]. The actual prevalence of HS is yet to be evaluated because it varies greatly between available reports. Studies report the prevalence from 0.09% among the German population up to even 4% in the population of young women [23,24,25]. The pathogenesis is still unclear; however, inflammation, genetic predisposition and bacterial propagation are the most often mentioned mechanisms [22].

The results of this study confirmed that HS is a burdensome disease and has a very large effect on patients’ QoL. The impairment of QoL was comparable with previously conducted studies. The mean total DLQI score was similar to those reported by Matusiak et al. [5] (12.7 ± 7.7 points), Frings et al. (12 ± 7.0 points) [26], Jørgensen et al. (11.9 ± 7.6 points) [27] and Kourins et al. (11.43 ± 6.61 points) [28]. However, there are also reports on much lower QoL impairment due to the disease (8.4 ± 7.5 and 8.9 ± 8.3 points) [29,30]. We believe that such differences are mostly due to distinctions in HS severity and duration. In the above-mentioned study by Onderdijk et al. [30], HS severity assessed with the Hurley staging system was lower than among our patients (13.5% vs. 17.2% for Hurley stage 3) [30]. Moreover, in remote questionnaire studies, it is impossible to assess the actual severity of the disease and, therefore, the actual impairment of QoL [29]. It is also worth emphasizing that some of the subjects of the study by von der Werth [29] had the disease inactive for 12 months, and 21 patients (18.4%) scored 0 points. This may have biased the DLQI scores downwards. The common use of the DLQI score allows us to compare the impact of HS with the effect of other dermatoses on QoL. The results of this study confirm prior results that the burden of HS is greater than those reported among other skin conditions, including psoriasis, chronic urticaria, atopic dermatitis, acne or alopecia [31,32,33,34,35]. In comparison to the above-mentioned dermatoses, which are believed to cause significant disability, the DLQI scores for HS patients are markedly higher, and this indicates that, to date. HS is the most distressing dermatological condition.

We found a significantly higher perceived impairment in QoL in female than male patients. This finding was previously reported by Kluger et al. [36] in a study with 26 HS patients. Similarly, in almost every DLQI item and domain, women scored significantly higher than men. We also observed a high correlation between pain and the DLQI, known to have a significant influence on daily functioning [37]. HS-associated pain could influence females more than males due to differences in neuroimmune pain modulation. It has been shown that due to multiple mechanisms (e.g., hormonal modulation), women suffer from pain more frequently than men and are more sensitive to it [38]. Moreover, according to studies regarding QoL impairment in different dermatoses (e.g., vitiligo, psoriasis or atopic dermatitis) [39,40,41], women tend to suffer more from skin-associated diseases. Zhang et al. [42] tried to explain this phenomenon and stated that, compared to men, women more frequently believe that physical appearance is important to their personal or social values. It may be also attributed to patients’ maladaptive assumptions about appearance and society’s focus on body and beauty [42]. Interestingly, Jørgensen et al. [27] did not confirm this finding in their study of 339 HS patients. The differences in our results and the above-mentioned study may indicate that although pain is important, there are other factors that influence patients’ quality of life. There are significant differences in psychological response to pain and chronic disorders between males and females. Certain coping strategies commonly used by women may be more effective with chronic pain and, therefore, diminish the difference in QoL impairment [43].

It has been well reported that obesity and smoking play an important role in the pathogenesis of HS [44]. Although the exact mechanism of smoking in HS is not well explained, a high expression of nicotine receptors was found on the follicular epithelium [45]. Regarding obesity, not only it is believed to be its own state of inflammation but also may increase skin folds’ friction, which is an important mechanism of HS development [46]. QoL was significantly lower among smokers and correlated weakly with the number of cigarettes per day. Similarly, the correlation between BMI and the DLQI was weak but significant. To the best of our knowledge, this is the second paper addressing the influence of smoking on patients’ QoL. Earlier this year, Jørgensen et al. [27] did not find any relationship. However, there are too few data on the topic to draw final conclusions.

The decrease in QoL may differ regarding the affected localization. It was previously reported that psoriatic patients with anogenital localization tend to have a significantly lower QoL [47]. Similarly, HS anogenital localization was linked to a higher burden and impairment of a person’s life [5,27]. However, our results are not consistent with the above-mentioned studies. Interestingly, the highest DLQI score, among our patients, was reported by those with neck involvement. We believe that, contrary to the anogenital area, which is not visible during the day, neck involvement could cause psychical distress on a daily basis. Moreover, due to the pain, tenderness and purulent flow, all head movement would be accompanied by physical and psychical discomfort.

We understand that our study has some limitations. As it concentrated on the baseline evaluation of HS patients who were selected for LAight therapy^®^, it may not reflect the whole population of HS patients. The use of only the DLQI and the lack of additional psychological instruments makes it impossible to assess associated psychological and psychiatric disorders (e.g., depression or anxiety). However, we believe that due to the studied sample size, which markedly exceeds the representative number of patients for the HS population, this study provides important insights into the HS-associated impairment of QoL.

## 5. Conclusions

In conclusion, to the best of our knowledge, this is the biggest study assessing the impairment of QoL in HS patients. Moreover, it presents a correlation with demographic data and clinical parameters. Our study highlights the enormous impact of HS on QoL. It is important to remember that, to date, HS seems to be a most burdensome dermatosis. Knowledge of QoL impairment in such patients is necessary for the proper understanding and holistic management of this disease.

## Figures and Tables

**Table 1:** Quality-of-life impairment assessed with DLQI (mean ± SD points).

DLQI	Total (n = 1795)	Female (n = 1152)	Male (n = 643)	Female vs. Male
DLQI Total	13.2 ± 8.1	14.2 ± 8.0	11.5 ± 8.0	**0.000000**
DLQI Item 1	1.7 ± 1.0	1.7 ± 1.0	1.5 ± 1.0	**0.000055**
DLQI Item 2	1.4 ± 1.2	1.5 ± 1.2	1.1 ± 1.1	**0.000000**
DLQI Item 3	1.1 ± 1.1	1.2 ± 1.1	1.0 ± 1.1	**0.001810**
DLQI Item 4	1.7 ± 1.2	1.9 ± 1.1	1.4 ± 1.2	**0.000000**
DLQI Item 5	1.4 ± 1.2	1.5 ± 1.2	1.3 ± 1.2	**0.001835**
DLQI Item 6	1.5 ± 1.3	1.6 ± 1.3	1.5 ± 1.3	0.147319
DLQI Item 7	1.1 ± 1.2	1.1 ± 1.1	1.1 ± 1.2	0.997689
DLQI Item 8	0.9 ± 1.1	1.1 ± 1.2	0.7 ± 1.0	**0.000000**
DLQI Item 9	1.2 ± 1.3	1.4 ± 1.3	0.9 ± 1.2	**0.000000**
DLQI Item 10	1.1 ± 1.1	1.2 ± 1.2	1.0 ± 1.1	**0.006825**
**DOMAINS**				
Symptoms and feelings	3 ± 1.8	3.2 ± 1.8	2.6 ± 1.7	**0.000000**
Daily activities	2.8 ± 2.0	3.1 ± 1.9	2.8 ± 2.2	**0.000000**
Leisure	2.9 ± 2.2	3 ± 2.2	2.4 ± 2.0	**0.010427**
Work and school	1.1 ± 1.2	1.1 ± 1.1	1.1 ± 1.2	0.997689
Personal relationships	2.2 ± 2.2	2.5 ± 2.3	1.6 ± 2.0	**0.000000**
Treatment	1.1 ± 1.1	1.2 ± 1.2	1 ± 1.1	**0.006825**

n—number of patients; DLQI—Dermatology Life Quality Index; bold—*p* < 0.05.

**Table 2:** Life impairment assessed with total DLQI regarding the affected lesions.

Affected Body Area	Singular	Multiple
Head (mean ± SD points)	14.9 ± 10.1 (n = 10)	13.3 ± 8.3 (n = 789)
Neck (mean ± SD points)	10 (n = 1)	**14.7 ± 8.3 (n = 129)**
Upper extremities (mean ± SD points)	11.3 ± 8.3 (n = 13)	13.3 ± 8.2 (n = 706)
Armpits (mean ± SD points)	12.28 ± 7.1 (n = 28)	13.3 ± 8.2 (n = 876)
Breast area (mean ± SD points)	15.1 ± 9 (n = 24)	13.3 ± 8.1 (n = 1213)
Anogenital area (mean ± SD points)	15.2 ± 7.1 (n = 16)	13.5 ± 8 (n = 1151)
Lower extremities (mean ± SD points)	9 ± 7.1 (n = 2)	**11.9 ± 7.9 (n = 347)**
Back (mean ± SD points)	7.3 ± 4.5 (n = 8)	12.5 ± 7.7 (n = 35)
Buttocks (mean ± SD points)	-	15.8 ± 10.9 (n = 4)

Bold—*p* < 0.05; n—number of patients; SD—Standard deviation.

## Data Availability

The data presented in this study are available on request from the corresponding author.
